# Plasma catecholamine levels in the acute and subacute stages of takotsubo syndrome: Results from the Stockholm myocardial infarction with normal coronaries 2 study

**DOI:** 10.1002/clc.23723

**Published:** 2021-09-07

**Authors:** Shams Y‐Hassan, Peder Sörensson, Christina Ekenbäck, Magnus Lundin, Stefan Agewall, Elin Bacsovics Brolin, Kenneth Caidahl, Kerstin Cederlund, Olov Collste, Maria Daniel, Jens Jensen, Claes Hofman‐Bang, Patrik Lyngå, Eva Maret, Nondita Sarkar, Jonas Spaak, Oscar Winnberg, Martin Ugander, Per Tornvall, Loghman Henareh

**Affiliations:** ^1^ Coronary Artery Disease Area, Heart and Vascular Theme Karolinska Institute and Karolinska University Hospital Stockholm Sweden; ^2^ Department of Medicine Solna, Karolinska Institutet, and Department of Cardiology Karolinska University Hospital Stockholm Sweden; ^3^ Division of Cardiovascular Medicine Karolinska Institutet, Department of Clinical Sciences, Danderyd Hospital Stockholm Sweden; ^4^ Department of Clinical Physiology Karolinska University Hospital, and Karolinska Institutet Stockholm Sweden; ^5^ Institute of Clinical Medicine University of Oslo Oslo Norway; ^6^ Department of Clinical Science, Division of Medical Imaging and Technology Intervention and Technology at Karolinska Institutet Stockholm Sweden; ^7^ Department of Radiology Capio S:t Görans Hospital Stockholm Sweden; ^8^ Department of Radiology Södertälje Hospital Södertälje Sweden; ^9^ Department of Clinical Science and Education Södersjukhuset, Karolinska Institutet, and Cardiology Unit, Södersjukhuset Stockholm Sweden; ^10^ Department of Clinical Science and Education, Södersjukhuset, Karolinska Institutet, and Department of Cardiology Capio St: Görans Hospital Stockholm Sweden; ^11^ Department of Cardiology Karolinska University Hospital Stockholm Sweden; ^12^ Kolling Institute, Royal North Shore Hospital, and Charles Perkins Centre, Faculty of Medicine and Health University of Sydney Sydney Australia

**Keywords:** catecholamines, metanephrines, myocardial infarction, neurogenic stunned myocardium, takotsubo

## Abstract

**Aims:**

It is well‐accepted that takotsubo syndrome (TS) is characterized by a massive surge of plasma catecholamines despite lack of solid evidence. The objective of this study was to examine the hypothesis of a massive catecholamine elevation in TS by studying plasma‐free catecholamine metabolites in patients participating in the Stockholm myocardial infarction (MI) with normal coronaries 2 (SMINC‐2) study where TS constituted more than one third of the patients.

**Methods and results:**

The patients included in the SMINC‐2 study were classified, according to cardiac magnetic resonance (CMR) imaging findings (148 patients), which was performed at a median of 3 days after hospital admission. Plasma‐free catecholamine metabolites; metanephrine, normetanephrine, and methoxy‐tyramine were measured on day 2–4 after admission. Catecholamine metabolite levels were available in 125 patients. One hundred and ten (88%) of the 125 patients included in SMINC‐2 study, and 38 (86.4%) of the 44 patients with TS had completely normal plasma metanephrine and normetanephrine levels. All patients had normal plasma methoxy‐tyramine levels. Fourteen (11.2%) of the 125 patients included in SMINC‐2 study, and 5 (11.6%) of the 43 patients with TS had mild elevations (approximately 1.2 times the upper normal limits) of either plasma metanephrine or normetanephrine. One patient with pheochromocytoma‐triggered TS had marked elevation of plasma metanephrine and mild elevation of plasma normetanephrine. There were no significant differences between the number or degree of catecholamine metabolite elevations between the different groups of patients with CMR imaging diagnosis included in SMINC‐2 study.

**Conclusion:**

There was no evidence of massive catecholamine elevations in the acute and subacute stages of TS apart from one patient with pheochromocytoma‐induced TS. Most of the TS patients had normal catecholamine metabolites indicating that blood‐borne catecholamines do not play a direct role in the pathogenesis of TS.

## 1 INTRODUCTION

Takotsubo syndrome (TS) is a well‐recognized acute cardiac disease entity with a clinical presentation resembling that of an acute coronary syndrome (ACS).^1^, ^2^ The term “takotsubo‐like” (tako = octopus, tsubo = a pot) was introduced by Sato and Dote in the early 1990s to describe the left ventricular shape during systole in patients presenting with clinical features of myocardial infarction (MI) but without obstructive coronary arteries.^3^, ^4^ The defining feature of TS is the reversible left ventricular wall motion abnormality (LVWMA), which has a characteristic circumferential pattern resulting in a conspicuous ballooning of the left ventricle during systole. The left ventricular dysfunction is incongruent with the coronary artery supply territory.^2^ The LVWMA may have mid‐apical, mid‐ventricular, mid‐basal, or focal left ventricular ballooning's pattern.^2^ Global left ventricular contractile abnormality has also been reported.^5^ The right ventricle is involved in about one third of patients with TS.^6^ A trigger stress factor (an emotional or a physical) may precede the onset of TS in about 70% of cases.^2^, ^7^ TS affects predominantly post‐menopausal women. Several pathophysiological mechanisms for the development of TS have been discussed. The main proposed mechanisms are myocardial ischemia, left ventricular basal hyperkinesis causing severe intraventricular pressure gradient, autonomic nervous system dysfunction with sympathetic hyper‐activation including local cardiac sympathetic disruption and norepinephrine seethe and spillover, blood‐borne catecholamine myocardial toxicity, and epinephrine‐induced switch in signal‐trafficking.^2^, ^8^, ^9^, ^10^, ^11^, ^12^, ^13^ According to the literature, one of the characteristic features of TS is a massive elevation of circulating plasma catecholamines.^2^, ^8^, ^14^ This is based on a study of 13 patients with emotional‐induced TS, which showed “massive elevation of plasma catecholamines and their metabolites” during the first 7–9 days after admission.^15^ However, a massive elevation of circulating plasma catecholamines has not been replicated in other studies.^16^, ^17^, ^18^, ^19^, ^20^ One of the disadvantages in measurement of plasma catecholamines is their short half‐lives where catecholamine elevations may be missed. The half‐lives of the plasma‐free catecholamine metabolites are longer than the parent catecholamines (epinephrine, norepinephrine, and dopamine) half‐lives, which are <5 min.^21^ The half‐life of plasma free metanephrine was reported to be 60–105 min and that of plasma free normetanephrine was 95 min.^21^ For this reason, measurement of plasma‐free metanephrines (metanephrine and normetanephrine) or urine total metanephrines has replaced measurement of plasma catecholamines as the recommended screening test for pheochromocytoma/paraganglioma. The aim of this study was to examine the hypothesis of a massive catecholamine elevation in TS through studying plasma‐free catecholamine metabolites in Stockholm MI with normal coronaries 2 (SMINC‐2) study where TS constituted more than one third of the patients.

## 2 METHODS

The plasma levels of catecholamine metabolites (metanephrine, normetanephrine, and methoxy‐tyramine) were measured in patients included in SMINC‐2 study. The SMINC‐2 study (Clinical Trials NCT02318498) was an open, prospective, nonrandomized, multi‐centre study with the aim to study patients with MI and nonobstructive coronary arteries (MINOCA) imaged with 1.5‐T cardiac magnetic resonance (CMR) with T1 and extracellular volume mapping early after hospital admission, compared to patients with MINOCA imaged using 1.5‐T CMR without mapping techniques from SMINC‐1 study as historic controls^22^, ^23^ . Consecutive patients from the five major hospitals in Stockholm, Sweden, 35–69 years old, fulfilling the existing diagnostic criteria of MI, with angiographically normal coronary arteries were included. The methods and the inclusion and exclusion criteria are discussed elsewhere.^23^, ^24^, ^25^ The plasma catecholamine metabolite levels were measured on day 2–4 after admission. All plasma‐free catecholamine metabolites were obtained using phlebotomy at rest in a supine position. Blood samples were placed on ice and immediately centrifuged, and the plasma was frozen. The measurements were made using high‐performance liquid chromatography. The upper normal limits (UNL) for fP‐metanephrine was <0.3 nmol/L, for fP normetanephrine, it was depending on the age of the patient, < 18 years <0.5 nmol/L, 18–29 years <0.6 nmol/L, 29–39 years <0.7 nmol/L, 39–49 years <0.8 nmol/L, 49–60 years <0.9 nmol/L, >60 years <1.1 nmol/L, and for fP methoxy‐tyramine, it was <0.2 nmol/L. The study was performed in accordance with the Declaration of Helsinki and Good Clinical Practice and was approved by the Stockholm Regional Board of Ethics (2014/131–31/1, 2014/1546–32). All patients provided written informed consent.

### 2.1 Statistical analysis

Results are reported as mean ± SD except where indicated otherwise. Kruskal‐Wallis H test was used for comparing independent samples of different sample sizes. Mann–Whitney U test was used to compare continuous variables and Fisher's exact test to compare categorical variables between the two groups with increased versus normal level of catecholamines. Box plot diagram was used to show plasma concentration of normetanephrine in the different subgroups of the patients. Statistical significance was taken at level of *p* < .05.

## 3 RESULTS

**FIGURE 1:**
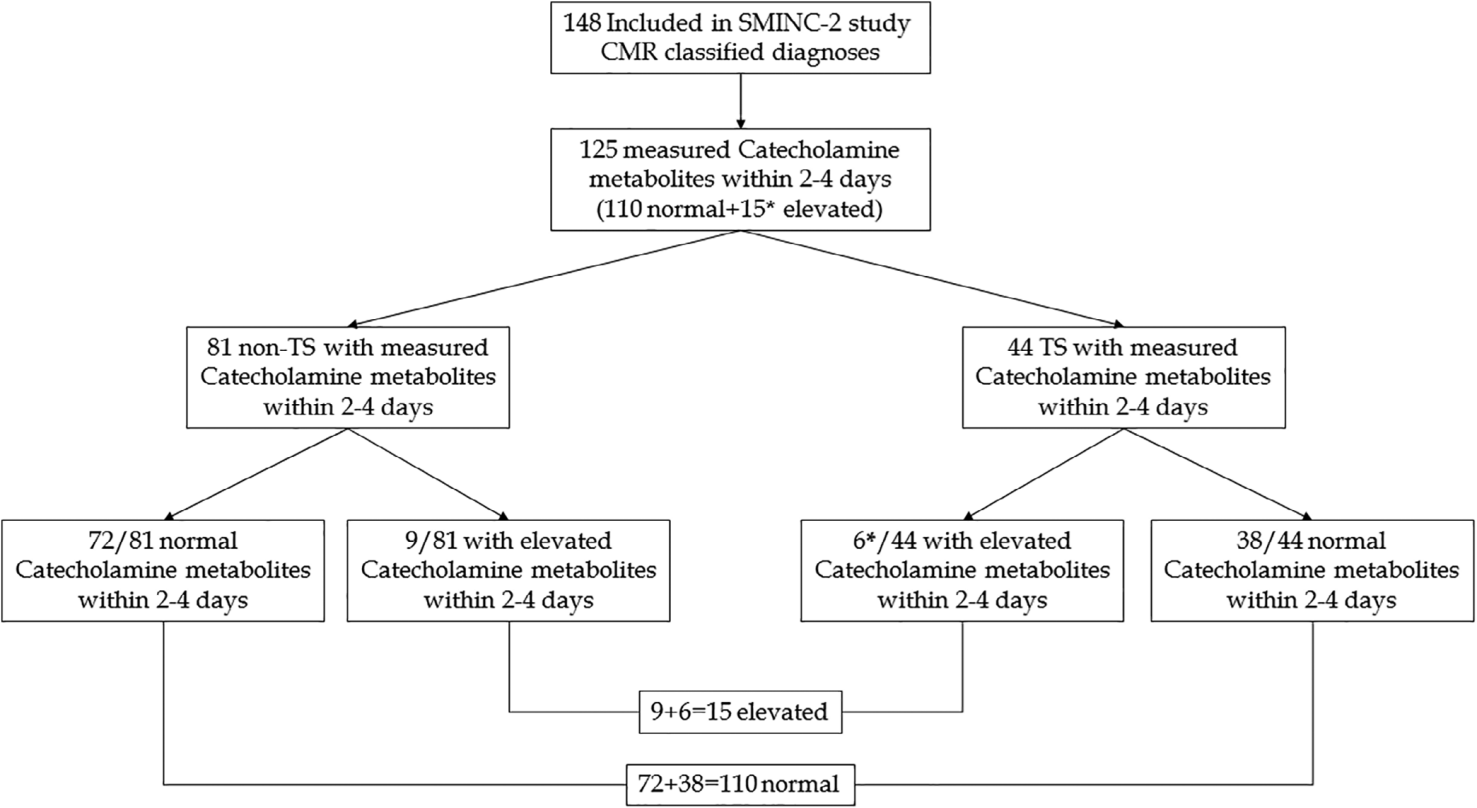
Flow chart illustrating the number of patients included in SMINC‐2 study with CMR diagnoses including TS group and the number of patients with normal or elevated plasma catecholamine metabolites in TS and non‐TS groups. *One of the patients had pheochromocytoma‐induced TS. CMR, cardiac magnetic resonance; TS, takotsubo syndrome

**FIGURE 2:**
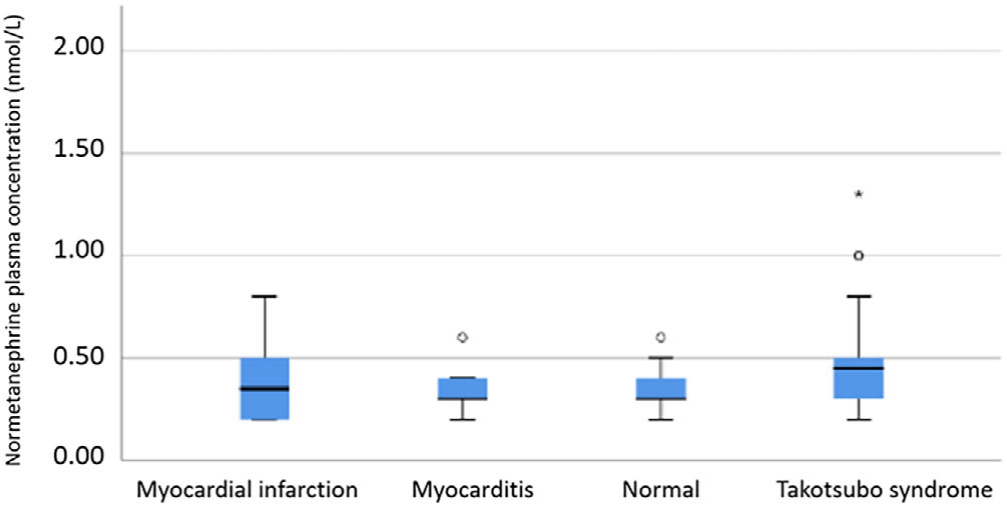
Box plot showing the distribution of plasma normetanephrine among the study groups

Flow chart illustrating the number of patients included in SMINC‐2 study with CMR diagnoses including TS group and the number of patients with normal or elevated plasma catecholamine metabolites in TS and non‐TS groups is shown in Figure 1. The baseline characteristics of the study patients diagnosed by CMR findings are shown in Table 1. Plasma‐free metanephrine, normetanephrine, and methoxy‐tyramine levels were available in 125 patients with CMR imaging diagnosis as shown in Table 2. The baseline characteristics in patients with elevated versus nonelevated level of catecholamine metabolites are shown in Table 3. The CMR definition of TS was according to the international takotsubo diagnostic criteria^2^ as apical, midventricular, basal or focal LVWMA with circumferential or segmental myocardial edema incongruent with the coronary artery supply territory, in the absence of corresponding late gadolinium enhancement. TS constituted 35% of the patients as shown in Figure 1. One hundred and ten (88%) out of 125 patients included in SMINC‐2 study had normal plasma‐free metanephrine and normetanephrine levels. All 125 (100%) patients had normal plasma methoxy‐tyramine levels. Ten (8%) (two in MI, three in TS, one in myocarditis, one in dilated cardiomyopathy [DCM], and three in normal groups) patients of 125 had mild elevation of plasma metanephrine (1.24 times the UNL). Four (3.2%) (one in MI, two in TS, and one in normal group) patients out of 125 had mild elevation of plasma normetanephrine (1.21 times the UNL). Only one patient had elevation of both plasma metanephrine (marked, 5.7 times the UNL) and plasma normetanephrine (mild, 1.45 times the UNL) (Table 2). This patient was later found to have pheochromocytoma‐induced TS. There were no differences in patient characteristics between patients with and without increased plasma‐free metanephrine and/or normetanephrine (Table 3). Thirty‐eight (86.4%) of the 44 patients with TS had completely normal plasma metanephrine and normetanephrine levels. Only five patients (11.6%) of 43 (one pheochromocytoma‐induced TS excluded) had mild elevation of either plasma metanephrine (three patients) or normetanephrine (two patients) (Table 2). As mentioned above, one patient with pheochromocytoma‐triggered TS had marked elevation of plasma metanephrine and mild elevation of plasma normetanephrine. Three of the patients with TS with mild elevation of catecholamine metabolites had mid‐ventricular TS pattern and two had classical apical TS pattern. The distribution of normetanephrine among the study groups is shown by boxplot diagram in Figure 2. The distribution of metanephrine and methoxy‐tyramin showed no variation among the study groups. There were no significant differences between the number or degree of catecholamine metabolite levels between the different groups of patients with CMR imaging diagnosis included in SMINC‐2 study.

**TABLE 1:** Baseline characteristics of the study patients diagnosed by CMR

	Normal *n* = 30	Takotsubo syndrome *n* = 44	Myocarditis *n* = 20	Myocardial infarction *n* = 26	CMP *n* = 5	*p* value

Age, years, mean ± SD	55 ± 9	60 ± 7	54 ± 8	55 ± 8	60 ± 9	0.257
Female sex, *n* (%)	18 (60)	41 (93)	11 (55)	17 (65)	3 (60)	.003
Smoking, *n* (%)	9 (30)	5 (11)	3 (15)	5 (19)	1 (20)	0.365
Family history of CAD, *n* (%)	6 (20)	16 (36)	8 (40)	4 (15)	1 (20)	0.188
Diabetes mellitus, *n* (%)	1 (3)	6 (14)	2 (10)	0	0	0.187
Treated hypertension, *n* (%)	2 (7)	15 (34)	3 (15)	3 (12)	0	0.017
Treated hyperlipidemia, *n* (%)	1 (3)	8 (18)	1 (5)	1 (4)	0	0.111
Psychiatric disease, *n* (%)	2 (7)	7 (16)	2 (10)	1 (4)	1 (20)	0.463
Thromboembolic disorders, *n* (%)	1 (3)	1 (2)	0	0	0	1.000
Normal ECG, *n* (%)	19 (63)	23 (52)	12 (60)	20 (77)	5 (100)	0.115
Max troponin, mean ± SD	90 ± 108	280 ± 222	278 ± 388	510 ± 387	174 ± 123	<.001
LVEF, mean ± SD	58 ± 5	55 ± 7	60 ± 6	57 ± 4	47 ± 17	.011
Migraine, *n* (%)	3 (10)	7 (16)	5 (25)	6 (23)	2 (40)	0.393
Chronic inflammatory disease, *n* (%)	1 (3)	3 (7)	1 (5)	0	0	0.694

Abbreviations: CAD, coronary artery disease; CMP, Cardiomyopathy; CMR, cardiac magnetic resonance; ECG, electrocardiogram; LVEF, left ventricular ejection fraction; SD, standard deviation.

*Note*: Data are presented as mean for continuous and *n* (percentage) for categorical variables.

**TABLE 2:** Results of plasma free catecholamine metabolites in SMINC‐2 study

CMR imaging diagnosis	Subgroups of diagnosis*N* = 148	Measured catecholamine metabolites in subgroups of patients*N* = 125	Patients with elevated metanephrine*N* = 11	Patients with elevated nor‐metanephrine*N* = 5	Patients with elevated methoxytyramine*N* = 0	Percentage of metanephrine and normetanephrine elevation
Myocardial infarction	32	26	2	1	0	11.5%
Takotsubo syndrome	52	44	3 + (1 Pheo)	2 + (1 Pheo)	0	11.6% (Pheochromocytoma case is excluded)
Myocarditis	25	20	1	0	0	5%
DCM	3	3	1	0	0	—
HCM	2	2	0	0	0	—
Normal	34	30	3	1	0	13.3%

Abbreviations: CMR, cardiac magnetic resonance; DCM, dilated cardiomyopathy; HCM, hypertrophic cardiomyopathy; MINOCA, myocardial infarction with normal coronary arteries; N, number; Pheo, pheochromocytoma; SMINC‐2, Stockholm Myocardial Infarction with Normal Coronaries 2.

*Note*: Normal reference values: fP‐metanephrine <0.3 nmol/L; fP normetanephrine depending on the age the patient, <18 years <0.5 nmol/L, 18–29 years <0.6 nmol/L., 29–39 years <0.7 nmol/L, 39–49 years <0.8 nmol/L, 49–60 years <0.9 nmol/L, >60 years <1.1 nmol/L; fP methoxy‐tyramine <0.2 nmol/L.

**TABLE 3:** Baseline characteristics in patients with elevated versus nonelevated level of catecholamine metabolites

	Elevated level of catecholamine metabolites. *N* = 15	Normal level of catecholamine metabolites. *N* = 110	*p value*

Age, years, mean ± SD	57 ± 9	57 ± 8	0.80
Female sex, *n* (%)	9 (60)	96 (87)	0.357
Smoking, *n* (%)	4 (27)	22 (20)	0.51
Family history of CAD, *n* (%)	4 (27)	36 (33)	0.77
Diabetes mellitus, *n* (%)	1 (7)	10 (9)	0.99
Treated hypertension, *n* (%)	2 (13)	26 (24)	0.52
Treated hyperlipidemia, *n* (%)	1 (7)	11 (10)	0.99
Psychiatric disease, *n* (%)	1 (7)	14 (13)	0.69
Thromboembolic disorders, *n* (%)	1 (7)	1 (1)	0.23
Normal ECG, *n* (%)	11 (73)	66 (60)	0.404
Max troponin, mean ± SD	338 ± 403	268 ± 289	0.776
LVEF, mean ± SD	54 ± 9	57 ± 7	0.45
Migraine, *n* (%)	4 (27)	20 (18)	0.49
Chronic inflammatory disease, *n* (%)	0	1 (1)	0.99

Abbreviations: CAD, coronary artery disease; CMR, cardiac magnetic resonance; ECG, electrocardiogram; LVEF, left ventricular ejection fraction; SD, standard deviation.

*Note*: Data are presented as mean for continuous and *n* (percentage) for categorical variables.

## 4 DISCUSSION

The main finding in this study was that most of the patients (88%) included in the SMINC‐2 study, being diagnosed with MI, TS, myocarditis, DCM, hypertrophic cardiomyopathy, or normal according to CMR imaging, had normal plasma catecholamine metabolites in the form of plasma‐free metanephrine and normetanephrine. All patients had normal plasma methoxy‐tyramine levels. Fourteen patients (11.2%) had mild elevations of either metanephrine or normetanephrine. Only five (11.6%) of 43 patients with TS had mild elevations (approximately 1.2 times the upper normal limits) of either plasma metanephrine or normetanephrine. One patient with TS had marked elevation of metanephrine, which was due to pheochromocytoma. Furthermore, there were no differences in the plasma levels of plasma‐free catecholamine metabolites between different groups of patients included in SMINC‐2 study.

### 4.1 Catecholamine levels in TS in other published studies

A massive surge of plasma catecholamines in patients with TS is stated in most of the literatures reporting on TS.^2^, ^8^, ^14^ This is based on one study reporting on 13 patients with emotional‐induced TS^15^ discussed below. In three other larger studies,^26^, ^27^, ^28^ plasma epinephrine and norepinephrine levels were moderately elevated in patients with TS. Marfella et al.^26^ reported a moderate elevation in plasma epinephrine and norepinephrine levels in 48 patients with TS during the subacute stage (two‐ to threefold of the normal values for epinephrine and less than twofold for norepinephrine).^20^ There was no information on plasma catecholamine metabolite levels in that study. In another study comprising 32 patients with TS, Christensen et al.^27^ also reported moderate elevation of plasma epinephrine and norepinephrine during the subacute stage (day 2–4). Worth to mention is that some patients had normal plasma epinephrine levels^29^ in the same study. In a systematic review of 108 patients with published TS, Dr. John E Madias^28^ reported that the blood catecholamines in the form of epinephrine, norepinephrine, and dopamine were normal, or mildly/moderately elevated, with only six patients showing marked elevations.

Several other studies have reported similar results as our study with normal or mild elevations of plasma metanephrine and normetanephrine.^16^, ^17^, ^18^, ^20^, ^30^, ^31^ Madhavan et al.^17^ reported normal levels of free fractionated metanephrine in 19 patients with TS and 10 patients with ST‐elevation MI (STEMI). Plasma normetanephrine was normal in 14 (73.7%) of 19 patients, the remainder had mild elevations of plasma normetanephrine. Y‐Hassan and Henareh^16^ reported normal plasma metanephrine in 23 (79%) out of 29 TS patients and mild‐moderately elevated values (2.5‐fold the UNL) in five patients (17%) whereas plasma normetanephrine was normal in 19 out of 29 patients (66%) and mildly elevated (1.8‐fold the UNL) in nine patients (31%). In one patient with pheochromocytoma‐triggered TS, the plasma metanephrine and normetanephrine were very high (41‐fold the UNL for metanephrine and 39‐fold for normetanephrine). Regarding plasma catecholamine metabolites, our results are in line with the results of both Madhavan et al.^17^ and Y‐Hassan et al.^16^ studies.

Madhavan et al.^17^ also studied twenty‐four‐hour urinary levels of fractionated catecholamines and metanephrines (day 1–3), which were normal in all patients with TS. The urine normetanephrines were mildly elevated in only two patients. The mean urinary levels (within the first three consecutive days after admission) of metanephrine and normetanephrine were normal in another study also comprising of 19 patients with TS triggered by a physical or an emotional trigger factor.^19^ On the other hand, the urinary metanephrines and normetanephrines were markedly elevated in pheochromocytoma/paraganglioma‐induced TS in the same study.

### 4.2 The study with massive catecholamine elevation in TS

The only study, which has reported on massive catecholamine elevations, including catecholamine metabolites metanephrine and nor‐metanephrine, day 1 or 2; day 3, 4 or 5, and day 7, 8, or 9 in patients with emotional‐triggered TS is that published by Wittstein et al.^15^ They compared plasma catecholamine levels in 13 patients with emotional‐induced TS with those in seven patients with Killip class III MI. They found that the plasma catecholamine levels were markedly higher among patients with TS than among those with Killip class III MI. This finding could not be reproduced by Madhavan et al. where plasma metanephrine and normetanephrine levels in 19 patients with TS were compared to those in 10 patients with STEMI. There were no differences in plasma catecholamine metabolites between the two groups of patients. The results in our study are in line with Madhavan et al's study^17^ where most of the patients with TS and MI had normal plasma catecholamine metabolite levels.

Worth to mention, the results of the study performed by Wittstein et al.^15^ have been critically scrutinized. Re‐analysis of that study revealed that the main reason for the reported massive elevation of circulating plasma catecholamines and their metabolites was attributed to the comparison of the values obtained in that study to the “published normal values” and not to the UNL of the same laboratory where the analysis was done.^20^ When comparing the results of catecholamine levels in Wittstein et al.^15^ study with other published normal values, there will be also only moderate elevation of plasma catecholamine^20^ levels in Wittstein et al's study comparable to the results measured by Marfella^26^ and Christensen.^27^ Consequently, no study has shown massive elevation of plasma catecholamines in TS apart from patients with other explanation for a massive catecholamine surge as pheochromocytoma/paraganglioma, or acute intracranial processes as subarachnoid hemorrhage.^10^, ^32^ Our sub‐study of SMINC‐2 study provides further support for the absence of massive elevation of plasma catecholamines in TS.

### 4.3 The implication of the results of SMINC‐2 study on the pathophysiology of TS

The external administration of catecholamines including both epinephrine and norepinephrine and the innate massive catecholamine elevations in disease conditions as in pheochromocytomas and paragangliomas are recognized triggers for TS^32^, ^33^, ^34^, ^35^, ^36^, ^37^, ^38^ most probably through activation of the sympathetic nervous system with disruption of cardiac sympathetic nerve terminals with norepinephrine seethe and spillover.^1^, ^10^ However, the normal plasma catecholamines seen in most of the patients with TS and only mild elevation in the remainder challenges the theory that blood‐borne catecholamines cause myocardial toxicity in TS. It also argues against the epinephrine‐induced switch in signal trafficking, which was hypothesized by Lyon et al.^39^ in 2008. They stated that high levels of circulating epinephrine trigger a switch in the intracellular signal trafficking from Gs (stimulatory) protein to Gi (inhibitory) protein signaling through B2 adrenoreceptors (B2ARs). The authors proposed that this change in signaling is negatively inotropic and that the effect is greatest at the apical myocardium explaining the apical ballooning seen in TS. Several findings in patients with TS challenges this hypothesis as follows: First, most of the patients with TS have normal plasma epinephrine or epinephrine metabolites as confirmed by our study. Second, almost half of the patients with epinephrine‐triggered (externally administered or innate elevation) TS have apical sparing pattern of TS.^32^, ^33^, ^34^, ^36^, ^40^ Third, the histopathological findings of hypercontracted sarcomeres and contraction band necrosis in TS argues against the inhibitory effect of epinephrine on the myocardium causing decreased contractility.^41^ However, regarding catecholamines and TS, two important points need to be emphasized. First, as discussed earlier, both external administration of catecholamines and the innate catecholamine elevations in pheochromocytoma/ paraganglioma may act as trigger factors for TS.^32^, ^33^, ^34^, ^35^, ^36^, ^37^, ^38^ Second, substantial evidences argue for a role of the sympathetic nervous system hyper‐activation in the pathogenesis of TS with local cardiac sympathetic hyper‐activation and disruption where norepinephrine churn and foam at the cardiac sympathetic nerve terminals ending in norepinephrine spillover.^1^, ^10^, ^13^ Elevated norepinephrine levels in the coronary sinus in patients with TS, suggesting increased local myocardial catecholamine release, has been reported.^18^ This results in myocardial stunning in a unique circumferential pattern typically regional following most probably cardiac sympathetic nerve distribution.^1^, ^9^, ^10^ Further support for this hypothesis is the signs of local cardiac sympathetic denervation in the hypokinetic/akinetic regions, evidence of chemical (catecholamine) myocarditis in the regions of myocardial stunning and the histopathological findings of contraction band necrosis.^1^, ^9^, ^10^

### 4.4 Limitations

One limitation is that although the half‐lives of the measured plasma catecholamine metabolites (1–2 h) are longer than the parent catecholamines (<5 min) half‐lives, the 1–2 h. Half‐life is relatively short, and we may thus have missed episodes of massive catecholamine elevations. On the other hand, Wittstein et al.^15^ reported massive plasma catecholamine elevations from day 1 of admission to day 7–9. In such a case, one may expect that the plasma catecholamines should be elevated at any time during the first week after admission considering that catecholamine metabolites were measured 2–4 days after admission in our study. Another limitation was that plasma‐free catecholamine metabolites were not measured in 23 of 148 patients with CMR imaging diagnosis because blood samples were not prepared appropriately.

## 5 CONCLUSION

Most of the patients (88%) in the SMINC‐2 study including TS patients, which constituted more than one third of patients, had normal plasma catecholamine metabolites. The remainder had only mild elevations with no difference between diagnosis groups. All patients had normal plasma methoxy‐tyramine levels. Consequently, excluding other causes of massive elevation catecholamines (which may trigger TS) as pheochromocytoma or paraganglioma, there is no evidence for a massive surge of plasma catecholamines in patients with TS.

The data that support the findings of this study are available from the corresponding author upon reasonable request.

## Data Availability

The data that support the findings of this study are available from the corresponding author upon reasonable request.
